# Cysteine Cathepsin Activity Regulation by Glycosaminoglycans

**DOI:** 10.1155/2014/309718

**Published:** 2014-12-21

**Authors:** Marko Novinec, Brigita Lenarčič, Boris Turk

**Affiliations:** ^1^Faculty of Chemistry and Chemical Technology, University of Ljubljana, Večna pot 113, SI-1000 Ljubljana, Slovenia; ^2^Department of Biochemistry, Molecular and Structural Biology, Jozef Stefan Institute, Jamova Cesta 39, SI-1000 Ljubljana, Slovenia; ^3^Centre of Excellence for Integrated Approaches in Chemistry and Biology of Proteins, Jamova Cesta 39, SI-1000 Ljubljana, Slovenia

## Abstract

Cysteine cathepsins are a group of enzymes normally found in the endolysosomes where they are primarily involved in intracellular protein turnover but also have a critical role in MHC II-mediated antigen processing and presentation. However, in a number of pathologies cysteine cathepsins were found to be heavily upregulated and secreted into extracellular milieu, where they were found to degrade a number of extracellular proteins. A major role in modulating cathepsin activities play glycosaminoglycans, which were found not only to facilitate their autocatalytic activation including at neutral pH, but also to critically modulate their activities such as in the case of the collagenolytic activity of cathepsin K. The interaction between cathepsins and glycosaminoglycans will be discussed in more detail.

## 1. Introduction

Cysteine cathepsins are members of the papain-like cysteine peptidase family [1]. Despite the fact that the eleven cysteine cathepsins found in man [2] represent only a small fraction of the human proteolytic repertoire, these enzymes have been attracting a lot of attention for their diverse roles in physiological and pathological processes that range from nonspecific protein turnover within the endolysosomal pathway to highly specialized functions in tissue homeostasis. A number of excellent reviews have been published recently, summarizing the structural and functional characteristics of cysteine cathepsins in health and disease [3–5].

**Figure 1:**
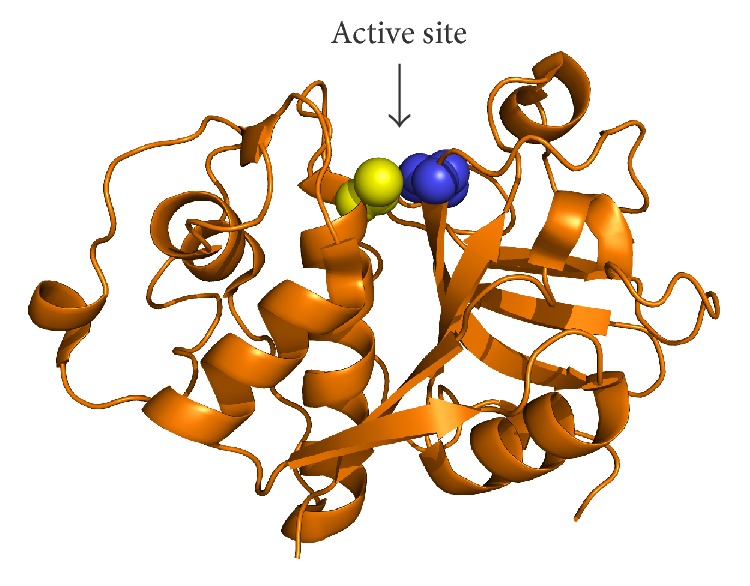
The papain-like peptidase fold illustrated on the crystal structure of papain. The protein is shown in cartoon representation and the position of the active site cleft is marked by an arrow. Catalytic residues Cys and His are shown as yellow and blue spheres, respectively. Coordinates were obtained from the Protein Data Bank under accession code 1PPN. The image was created with PyMOL (Schrödinger, LLC, Portland, OR, USA).

All the cathepsins share the same structural scaffold, also called the papain-like fold. The structure consists of two subdomains which have been termed the L- and R-domains referring to their position when the molecule is shown in the standard orientation (Figure 1). The active site cleft is at the top of the molecule between the L- and R-domains and contains the conserved catalytic dyad Cys-His (marked by yellow and blue spheres in Figure 1, resp.). In general, papain-like peptidases can act as endo- or exopeptidases. In a typical endopeptidase the primary specificity determinant is the S2 site [6] and well-determined sites on the enzyme interact with residues P3 through P2′ of the substrate [7]. Five of the eleven human members of the family (cathepsins F, K, L, S, and V) are exclusively endopeptidases, cathepsin B is also a peptidyl dipeptidase, cathepsin X is a carboxypeptidase, cathepsin H is an aminopeptidase, and cathepsin C is a dipeptidyl peptidase. The proteolytic activity of the remaining two members, cathepsins O and W, remains to be determined [4]. Most cysteine cathepsins are ubiquitously expressed in the human body, while some (cathepsins K, S, V, and W) are expressed in more restricted patterns [3]. Cathepsin K is abundantly expressed in osteoclasts and synovial fibroblasts [8, 9] but is also found in other cells of the hematopoietic, epithelial, and fibroblast lineages [10]. Highest expression levels of cathepsin S are found in antigen-presenting cells [11], cathepsin V is expressed predominantly in thymus and testis [12], and the expression of cathepsin W is restricted to CD8+ lymphocytes and natural killer cells [13].

## 2. Regulation of Cysteine Cathepsins Activity

Zymogen activation is one of the major means of regulations of cathepsin activity. All the cathepsins are namely synthesized as inactive zymogens and activated in the acidic milieu of the endolysosomal vesicles. The molecular mechanism of their activation was puzzling for a long time. The critical information came from the combination of structural studies of procathepsins B, K, and L, which showed that the propeptide runs through the active site of cathepsins in the opposite direction of the substrate, thus excluding the cleavage of the propeptide in the molecule without enormous and energetically unfavorable structural movements of the propeptide [15–19], thereby eliminating the unimolecular mechanism initially suggested, and detailed kinetic studies, which clearly demonstrated that the activation of cathepsin B is a bimolecular process [20]. The current model, which is mostly based on the cathepsin B studies, suggests that the propeptide in the cathepsin zymogen switches between two conformations, the so-called “closed” and “open.” In the “closed” conformation, favored at neutral to slightly acidic pH, the propeptide blocks the active site and prevents substrate hydrolysis, whereas, in the “open” form, favored at acidic pH below pH 5.0, the propeptide is removed from the active side cleft, resulting in a low catalytic activity of the zymogen. This activity is sufficient to activate another cathepsin zymogen in one or several steps, thereby starting the chain reaction, where such fully active mature cathepsin B processes the majority of zymogen molecules [21].

The other major regulators of cysteine cathepsins are macromolecular inhibitors that bind into the active site and thereby prevent association of the peptidase with its substrate. They belong to several distinct families including the cystatins, the thyropins, and the serpins that can, in addition to serine proteases, also inhibit several cathepsins [3, 22–25].

## 3. Glycosaminoglycans as Major Regulators of Cysteine Cathepsin Activity 

Glycosaminoglycans (GAGs) are heteropolysaccharides composed of repeating disaccharide units with a high negative charge. This is a result of the presence of multiple carboxyl groups and sulfate substitutions. Most of GAGs are sulfated, including chondroitin sulfates (CS), keratan sulfate (KS), dermatan sulfate (DS), heparan sulfate (HS), and heparin, whereas hyaluronan (HA) is the only nonsulfated GAG. In recent years GAGs have been emerging as important regulators of cysteine cathepsins with diverse effects on their targets. Traditionally, cysteine cathepsins had been viewed as lysosomal proteases and, like other lysosomal enzymes, had been known to be inhibited by intralysosomal GAGs in the resting lysosome [26]. Today, however, cysteine cathepsins are established as major players in extracellular proteolysis [27]. Their action in the glycosaminoglycan-rich extracellular environment raised questions about the interplay between cysteine cathepsins and GAGs outside of the lysosome. The two groups of endogenous human cysteine cathepsins most commonly associated with extracellular proteolysis are cathepsin L-like proteases (cathepsins K, L, S, and V in humans) and cathepsin B [27]. Data accumulated over the past two decades shows that the interplay between these peptidases and GAGs goes both ways; cysteine cathepsins are capable of cleaving proteoglycan core proteins and thereby release GAGs from their support, whereas GAGs in turn affect both the activity and stability of cysteine cathepsins in the extracellular space.

The regulation of papain-like cysteine peptidases by GAGs was first described for cathepsin L [28, 29]. In these early works it was found that GAGs and various negatively charged surfaces substantially accelerate activation of the cathepsin L zymogen into the mature form, including at pH close to the neutral, such as also found in the extracellular milieu in various disease conditions. This has been confirmed for several other cathepsins, the most important being cathepsins B and S [30, 31], and even for a* T. congolense* parasite homologue congopain [32], suggesting that GAGs and other negatively charged surfaces may play a major role in extracellular cathepsin activation in disease. However, recent findings with cathepsin S at high GAG concentration suggest that this enzyme may behave somewhat differently under such conditions with chondroitin-4-sulfate (C4S) even exhibiting a decelerating effect [33]. Nevertheless, facilitating autocatalytic activation of cathepsins by the negatively charged polysaccharide dextran sulfate also became a routine method in preparation of recombinant cathepsins [20, 34–36].

The majority of information about the molecular mechanism of GAG-assisted cathepsin activation came from a study by Caglič et al. using human cathepsin B as a model [31]. As shown, GAGs seem to contribute to the processing in two ways. First, upon binding they seem to convert the cathepsin zymogen into a better substrate. Second, binding of GAGs apparently favors the open conformation of the zymogen, thereby promoting activation not only at acidic pH but also at pH values closer to neutral. This seems to be the case for most GAGs and does not depend critically on the charge density of GAGs, as also HA was able to accelerate the activation, although to a lower extent, which is unusual for a protein-GAG interaction. Moreover, already a tetrasaccharide was sufficient for a prominent acceleration of cathepsin B autoactivation, which is substantially smaller than found for a number of other GAG-mediated reactions [37, 38]. The interaction is mediated by ionic interactions; however, there seems to be no conserved GAG-binding surface on the zymogens as completely unrelated residues in procathepsins L and B, which were largely located on the prodomains, were found to govern the interaction [31, 39].

The other important role of GAGs in the regulation of cathepsin activity came from the studies on papain, the archetypal representative of the family [40]. This mode of regulation quickly received more attention with the discovery that chondroitin-sulfate from cartilage prominently increased the collagenolytic activity of cathepsin K [41]. This particular peptidase had been discovered a few years prior as the sole protease responsible for collagen degradation in bone remodeling and immediately recognized as a potential drug candidate for the treatment of metabolic bone diseases, such as osteoporosis [42]. The interaction of cathepsin K with chondroitin-sulfate and other glycosaminoglycans was later examined in detail from both the structural and functional perspectives and glycosaminoglycans have been recognized as the first known allosteric regulators of a cysteine cathepsin peptidase [43, 44], as described in detail in the following sections. In parallel, functionally relevant interactions with glycosaminoglycans have also been documented for other members of the cysteine cathepsin family. Taken together, glycosaminoglycans have been found to affect both the activity and the stability of cysteine cathepsins. Kinetic profiles are usually consistent with hyperbolic mechanisms, indicating interactions with enzymes outside of the active site, possibly via allosteric mechanisms. The stabilizing effect is important especially because of the relative instability of cysteine cathepsins at neutral pH found in the extracellular matrix.

## 4. Regulation of Cathepsin K Activity and Stability

Of all papain-like peptidases, cathepsin K has been established as the cathepsin most tightly linked to glycosaminoglycans. It was originally identified as a protease expressed predominantly in osteoclasts [8] and its impaired activity was shown to result in severe bone disorders [42, 45]. Cathepsin K is a collagenase with unique activity among mammalian peptidases [46–48], which is specifically modulated by glycosaminoglycans via allosteric mechanisms [41, 44]. Due to its central role in bone turnover, cathepsin K is currently considered one of the most promising targets for the treatment of osteoporosis [49]. Apart from bone remodeling, cathepsin K is involved in diverse physiological and pathological processes (for a recent review see [10]). It can cleave a number of extracellular substrates, including proteoglycans, to release active glycosaminoglycans [50], which in turn modulate its activity. In cartilage, cathepsin K degrades both type I and type II collagens and thereby contributes to the development of various inflammatory joint diseases [9, 51, 52]. Moreover, cathepsin K has been associated with cardiovascular diseases, obesity, schizophrenia, and cancer [10].

The interaction of cathepsin K with different glycosaminoglycans has been the subject of in-depth investigation. Though several aspects of these interactions remain elusive, accumulated data suggest that the interactions are heterogeneous and diverse and probably involve multiple binding sites on the enzyme. Chondroitin-4-sulfate (C4S) was initially identified as the GAG with the most dramatic effect on cathepsin K [41] while the effects of chondroitin-6-sulfate (C6S), dermatan sulfate (DS), and hyaluronan (HA) were weaker. All tested GAGs increased the stability of cathepsin K over a broad pH range. C4S had the most prominent effect on the degradation of types I and II collagens by cathepsin K [41], whereas its effect on the hydrolysis of a synthetic substrate was virtually identical to that of C6S and DS and resulted in a twofold increase in the values of the specificity constant (*k*_cat_/*K*_*m*_). In a later study, keratan sulfate (KS) and C6S were found to have a stimulatory effect on cathepsin K similar to that of C4S, whereas heparin and HS had a limited effect on the collagenolytic activity of cathepsin K [53]. Early experiments have also suggested that complex formation with CS is necessary for the collagenolytic activity of cathepsin K [54]; however, recent findings have shown that type I collagen can also be efficiently degraded in the absence of glycosaminoglycans [55]. Nonetheless, bone-resident GAGs have been shown to potentiate the collagenolytic activity of cathepsin K and endogenous GAG concentrations in bone were sufficient for a maximal effect on cathepsin K activity [55].

The kinetic mechanism of the effect of CS, DS, and heparin (HP) on cathepsin K was also investigated in detail at physiological plasma pH of 7.4. Under these conditions, CS and DS were characterized as nonessential activators with a predominant effect on the affinity for the substrate *K*_*m*_ [44]. DS was more effective than CS, which was attributed to its greater flexibility due to fewer intramolecular hydrogen bonds [56]. Intrinsic fluorescence indicated that binding of GAGs affects the conformation of cathepsin K. In contrast to experiments performed at pH 5.5, CS and DS acted as inhibitors of collagen degradation at physiological plasma pH. In contrast, the kinetic mechanism of heparin was biphasic, indicating interaction with two distinct sites on the enzyme. Altogether, heparin was a strong activator of cathepsin K at physiological plasma pH, increasing both its collagenolytic and elastinolytic activities. Moreover, heparin had a strong stabilizing effect on cathepsin K under these conditions, resulting in a more than 5-fold increase in the half-life of the enzyme [44].

## 5. Structural Basis for the Interaction between Cathepsin K and GAGs

**Figure 2:**
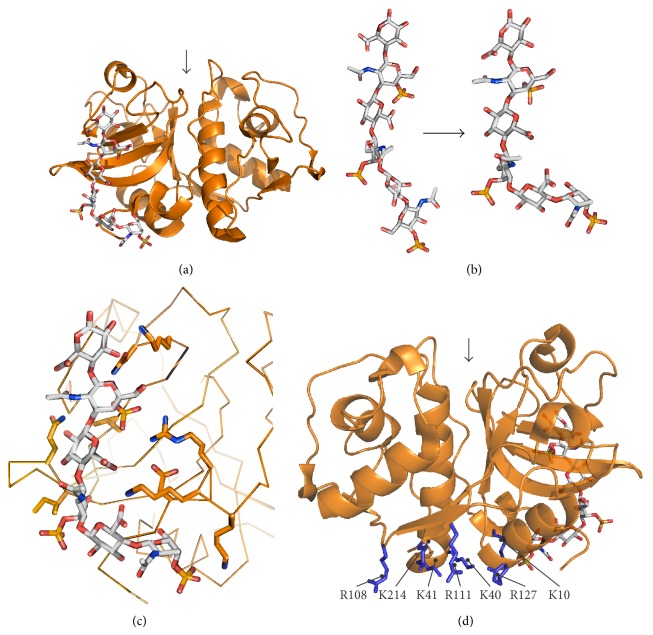
Interactions between human cathepsin K and GAGs. (a) Crystal structure of the cathepsin K/chondroitin-4-sulfate (C4S) complex. The protein is shown in cartoon representation and C4S is shown as sticks. (b) Conformational change in C4S upon binding to cathepsin K. (c) Detailed representation of the interaction in panel (a). C4S is shown as sticks. The backbone of cathepsin K is shown as ribbons and residues that interact with C4S are shown as sticks. (d) Location of the predicted second heparin-binding site in cathepsin K. Positively charged residues proposed to interact with heparin are shown as blue sticks. For orientation, C4S bound at the first binding site is shown as sticks. The position of the active site cleft is marked by an arrow. Coordinates of the cathepsin K/C4S complex were retrieved from the Protein Data Bank under accession code 3C9E. The solution structure of C4S was modeled using data from [14]. All images were created with PyMOL.

The crystal structure of cathepsin K and C4S revealed the structural basis for the interaction [43]. The binding site is located on the back of cathepsin K and interacts with three disaccharide units of CS in the crystal structure (Figure 2(a)). As usual, the glycosaminoglycan/protein interaction is mediated mostly by electrostatic interactions between the negatively charged GAG chain and positively charged residues on the enzyme. Binding of chondroitin-sulfate does not cause significant conformational changes in cathepsin K compared to the CS-free cathepsin K. Conversely, the CS chain is bent upon binding to cathepsin K (Figure 2(b)). The bulk of the conformational change can be attributed to the interaction with a short helical region Arg8-Lys9-Lys10 which interacts with four negatively charged groups on CS (Figure 2(c)). Further close contacts include Asp6, Ile171, Gln172, Asn190, Lys191, and Leu195 and a few additional water-mediated contacts [43].

The kinetic behavior of DS was analogous to that of CS: it was hence proposed that it interacts with cathepsin K in the same manner as CS [44]. Heparin, on the other hand, was proposed to bind to two sites on cathepsin K according to its kinetic profile. While the first binding site was proposed to be identical with that for CS/DS, the second binding site was predicted on the bottom of the molecule by chemical cross-linking experiments and computational modeling [44]. From a structural perspective, the predicted binding site is a continuation of the CD/DS-binding site and consists of several basic residues (Lys10, Lys40, Lys41, Arg108, Arg111, Arg127, and Lys214) organized in a ring-shaped structure (Figure 2(d)). Kinetic experiments have confirmed that heparin can be bound to both sites at the same time [44]. However, it remains to be determined whether this requires one HP chain that interacts with both sites at the same time or two separate HP chains. This is neither clear for the interaction of other GAGs with the second HP-binding site.

## 6. Interactions of GAGs with Cathepsins S and B

**Figure 3:**
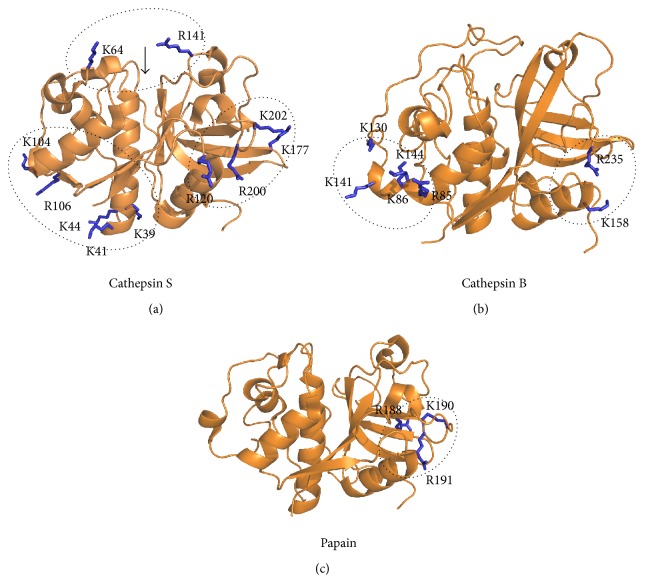
Predicted GAG-binding sites in papain-like peptidases. (a) Three predicted CS-binding sites in cathepsin S. (b) Two predicted HS/HP-binding sites in cathepsin B. (c) The conserved GAG-binding motif in papain. Predicted sites are shown in circles and positively charged residues at each site are shown as blue sticks and labeled. The position of the active site cleft is marked by an arrow. All coordinates were obtained from the Protein Data Bank (accession codes: 1NQC for cathepsin S, 3AI8 for cathepsin B, and 1PPN for papain, resp.). All images were created with PyMOL.

Apart from cathepsin K, two other human papain-like peptidases, cathepsins S and B, have been shown to be regulated by glycosaminoglycans in their mature forms [33, 57]. Cathepsin S, the closest relative of cathepsin K, is unusual among cysteine cathepsins for being stable at neutral pH [58]. Cathepsin S has major physiological roles in antigen-presenting cells as the most important protease in antigen processing [59–61] and was recently found to be regulated by C4S [33]. In contrast to the activation effect observed with cathepsin K, C4S acted as an inhibitor of type IV collagen degradation by cathepsin S. Inhibition was also observed with HS, whereas HP, DS, C6S, and HA slightly increased the proteolytic activity of cathepsin S using type IV collagen as the substrate. C4S, C6S, and HS also inhibited the hydrolysis of Z-Phe-Arg-AMC via a partial, mixed mechanism. Similar to cathepsin K, subtle conformational changes in cathepsin S were observed upon C4S binding by intrinsic fluorescence spectroscopy. Three binding sites for C4S were predicted on cathepsin S by molecular docking (Figure 3(a)). One of the proposed sites is the active site, which, however, is not in agreement with the observed mixed inhibition profile of C4S; the second is located on the bottom right side of the molecule and corresponds to the recently identified allosteric site in cathepsin K [62], while the third is located at the bottom of the molecule and roughly corresponds to the secondary heparin-binding site identified in cathepsin K [44].

Cathepsin B is unique among cysteine cathepsins for being both an endopeptidase and a peptidyl dipeptidase, depending on the conformation of the occluding loop, a cathepsin B-specific structure that provides a pH-specific switch between both activities [63]. In the lysosome, the low pH restricts the protease to a closed, exopeptidase conformation, whereas the near-neutral pH of the extracellular environment promotes endopeptidase activity of cathepsin B [63]. Extracellular cathepsin B is most commonly associated with cancer and various types of arthritis [64, 65]. The protease localized to the cell surface in several studies and was found to be involved in cell migration under both physiological and pathological conditions [66, 67]. At the molecular level, it was shown to cleave a number of extracellular substrates, including laminin, type IV collagen, and fibronectin [68, 69]. Recently, cathepsin B has also been suggested to be a *β*-secretase that produces amyloid *β* peptides in secretory vesicles of neuronal chromaffin cells [70]. However, it has also been shown to degrade amyloid deposits in an animal model [71] and the overall outcome has been suggested to be determined by the balance between cathepsin B and its endogenous inhibitor cystatin C [72].

Binding of HP or HS has been shown to increase the stability of the otherwise unstable enzyme at alkaline pH (8.0), while slightly reducing the activity of the enzyme along the entire pH profile of the enzyme [57]. Computational simulations have predicted that heparin stabilizes the conformation of the molecule under these conditions and have predicted two putative GAG-binding sites (Figure 3(b)), one on each side of the enzyme [73]. The putative binding site in the L domain consists of five basic residues (Arg85, Lys86, Lys130, Lys141, and Lys144), while the one in the R domain contains only two (Lys158 and Arg235). The authors have suggested that the binding site in the R domain has higher affinity for shorter GAG fragments, such as the heparin disaccharide used in their docking simulations, whereas the one in the L domain is likely more relevant for the binding of longer GAG fragments [73].

## 7. Interactions of GAGs with Other Papain-Like Peptidases

Interestingly, papain has also been shown to interact with GAGs. Despite bearing no physiological relevance, these interactions point to evolutionarily conserved mechanisms of regulation within the family. HP inhibited papain by a hyperbolic mixed mechanism and affected its conformation [40]. A classical heparin-binding consensus sequence was identified in papain, in the form of the sequence 187-Ile-Arg-Ile-Lys-Arg-Gly-192. Structurally, this sequence is located on the right side of the molecule (Figure 3(c)) in a region that lies between both allosteric sites known in cathepsin K.

In addition, a few examples of proteases from protozoan parasites have been described that interact with GAGs, suggesting the possibility of their influence on host-parasite interactions. The cathepsin L homolog brucipain, a crucial virulence factor of the protozoan parasite* Trypanosoma brucei*, has been found to be allosterically modulated by HS. The effect of HS in this study was subtle and it had the ability to reverse substrate inhibition by a small dipeptide substrate (Z-Phe-Arg-AMC) [74]. A stronger effect of HS was observed for cruzipain from the related parasite* Trypanosoma cruzi*. In this case, HS was an activator of the peptidase that caused a significant (up to 6-fold) increase in the activity of the peptidase measured with a synthetic substrate. Moreover, HS increased the release of kinin from high molecular weight kininogen by cruzipain* in vitro* as well as by living trypomastigotes and reduced the inhibitory properties of kininogen towards cruzipain [75]. Similarly, HP was recently shown to modulate the activity of the cathepsin L-like peptidase rCPB2.8 from* Leishmania mexicana* [76]. In this case, HP and HS, but not CS or DS, inhibited the hydrolysis of Z-Phe-Arg-AMC by a hyperbolic mixed mechanism and affected the conformation of the protein [76]. Altogether these examples show that interactions with GAGs are not restricted to endogenous cysteine cathepsins but can also play diverse roles in host-pathogen interactions and can act either as part of the body's defense against invading pathogens or as factors contributing to the invasive mechanisms of the pathogen.

## 8. Pharmacological Targeting 

Cathepsin K currently represents the most attractive drug target among the cathepsins, although cathepsin S is also a relevant target in diseases associated with elevated immune response, such as bronchial asthma and psoriasis [5, 27]. Several cathepsin K inhibitors are currently under development, which target the active site of the enzyme (collected in [10, 27]). At the moment, the most promising inhibitor is odanacatib (Merck & Co., Inc., Whitehouse Station, NJ, USA), a nitrile warhead-containing inhibitor, highly selective for cathepsin K [77]. Phase III clinical trials for odanacatib have been successfully concluded and applications for approval are expected to be filed soon. If approved, the drug will position itself in the market against other new generation drugs, such as the anti-RANK-ligand antibody denosumab (Amgen, Inc., Thousand Oaks, CA, USA) and the teriparatide, a recombinant form of parathyroid hormone (Eli Lilly and Company, Indianapolis, IN, USA), as well as the well-established bisphosphonates [78]. Targeting the cathepsin K/chondroitin-sulfate interaction would represent an alternative to these treatments. Endogenous chondroitin-sulfate is sufficient to exhibit a maximum activation effect on cathepsin K and its digestion reduces the activity of cathepsin K by 40% [55]. A reduction in bone turnover of this magnitude would likely suffice for the treatment of patients with less severe bone density reduction. An added benefit would be that cathepsin K activity* per se* as well as the viability and cell count of osteoclasts and osteoblasts would remain undisturbed.
